# S01-1: A systems-based approach to physical activity in Scotland: A framework for national and local action

**DOI:** 10.1093/eurpub/ckae114.197

**Published:** 2024-09-26

**Authors:** Flora Jackson

**Affiliations:** Public Health Scotland

## Abstract

**Purpose:**

To share learning from the Public Health Scotland development and application of a pragmatic, evidence-driven, systems-based approach to physical activity policy in Scotland at a national and local level.

**Development:**

Public Health Scotland led the development of A Systems-based Approach to Physical Activity in Scotland: A Framework for National and Local Action. This involved translating the WHO Global Action Plan for Physical Activity (GAPPA) and ISPAH Eight Investments That Work for Physical Activity into a Scottish context and the facilitation of workshops, engaging 80 stakeholders from 47 organisations with a role in the physical activity system.

Through these workshops the approach was tested and refined, core strategic delivery outcomes and associated actions were identified, and a strategic outcomes framework and process developed, that can be used to direct physical activity policy development within national and local government.

Underpinned by the core principles of public health, this approach uses methods familiar to policymakers and practitioners including outcomes focused planning, evidence-based knowledge into action and quality improvement methodologies that operate alongside aspects of systems thinking.

**Implementation and dissemination:**

Adopted by Scottish Government and national partners, the approach has been used collaboratively to develop a new Physical Activity for Health Strategy for Scotland and revise the previously acclaimed Active Scotland Outcomes Framework devised to monitor, evaluate and review progress.

Working in partnership, sportscotland and Public Health Scotland are supporting local government partners apply the systems-based approach, to guide the development of local and regional evidence-based physical activity strategies and action plans.

**Evaluation:**

Implementation is monitored and evaluated via governance structures, national surveillance indicators, process indicators and an ongoing cycle of quality improvement and policy review.

**Conclusions and implications for policy and practice:**

This approach has influenced and guided the development of national and local physical activity strategies and actions plans that embrace the evidence that works to increase population levels of physical activity and reduce inactivity.

